# A Comparison of Mini Open Transverse Incision and Longitudinal Incision for the Release of Moderate and Severe Carpal Tunnel Syndrome

**DOI:** 10.7759/cureus.56677

**Published:** 2024-03-21

**Authors:** Georges F Bassil, Joeffroy Otayek, Ramzi C Moucharafieh, Mohammad Badra

**Affiliations:** 1 Department of Orthopedic Surgery, Lebanese University Faculty of Medicine, Beirut, LBN; 2 Department of Orthopedics, Lebanese American University Medical Center, Beirut, LBN; 3 Department of Orthopedics and Traumatology, Clemenceau Medical Center, Johns Hopkins Medicine International, Beirut, LBN; 4 Department of Orthopedic Surgery and Traumatology, Clemenceau Medical Center, Johns Hopkins Medicine International, Beirut, LBN

**Keywords:** carpal tunnel syndrome, transverse incision, longitudinal incision, mini-invasive release, neuroplasty, ctr

## Abstract

Objectives: Carpal tunnel syndrome is a common condition seen in daily clinical practice. Multiple minimally invasive techniques have emerged in the last decades for median nerve decompression. However, many research are needed to study the outcome on the patients and their safety profile.

Methods: We will compare group A that includes patients operated on using the minimally invasive transverse incision (number of patients = n = 221, females 76.7% and males 22.8%) versus group B that includes patients operated on using the longitudinal incision (n = 194, female 70.1% and male 29.9%) in term of clinical satisfaction and safety. The mean age of group A is 58.1±5.1 and that of group B is 58.8±4.8. The male and female distribution in both groups and the mean age were both similar with no statistically significant difference for the age (p = 0.79) or the gender distribution (p = 0.1). Data collected prospectively at regular intervals in time (preoperatively and at one month, three months, and six months post-carpal tunnel release (CTR)) between January 2006 and December 2021 were reviewed retrospectively. Patients’ clinical findings, grip strength measurement using a hand dynamometer, and postoperative satisfaction measured using the BCTQ (Boston Carpal Tunnel Syndrome Questionnaire) scoring system were recorded and analyzed for each technique.

Results: A total of 415 patients were included in our study. All patients included had moderate to severe median nerve compression documented by nerve-conducted studies with positive Tinel’s and Phalen’s signs. Baseline demographics between group A (CTR through a longitudinal palmar mini-incision) and group B (CTR with a mini-transverse incision at the palmar crease) didn’t show a statistically significant difference. Both groups showed improved grip strength and BCTQ scores at the post-operative follow-up.

Conclusions: Median nerve decompression using both types of incisions has resulted in the same functional outcomes and patient satisfaction.

## Introduction

Carpal tunnel syndrome (CTS) is the most common compressive focal mononeuropathy seen in clinical practice accounting for 90% of all entrapment neuropathies [1]. It occurs among individuals between 40 and 60 years of age and is three times more common in women than in men [2], with an incidence of 2-3% in the general population [3]. CTS results from compression of the median nerve that passes through the carpal tunnel. ﻿Patients mainly complain of painful paresthesia or burning pain in the thenar region of the hand that aggravates at night [4]. Surgical division of the transverse carpal ligament is the well-accepted surgical technique for the release of the median nerve compression once conservative management fails. Various techniques have been used to try to minimize the incision while giving the best outcome [5-7]. These modalities include conventional open CTR (carpal tunnel release) [5], limited incision CTR [6], and endoscopic CTR [7]. This study aims to ﻿compare functional outcomes, and symptoms severity of CTR using the limited palmar longitudinal incision and mini-open transverse flexor crease incision technique using routine surgical instruments.

## Materials and methods

We conducted a study at the Clemenceau Medical Center (CMC) hospital with the aim of comparing two mini-open CTR techniques: the transverse incision and the longitudinal incision. This comparison focused on assessing patient satisfaction and clinical outcomes during the six months following surgical intervention. Data were prospectively collected between January 2006 and December 2021 at regular intervals (pre-operative, one month, three months, and six months post-operative) and subsequently reviewed retrospectively. Ethical approval was granted by the Institutional Review Board (IRB) of CMC Hospital, with approval number 146372. Informed consent was obtained from patients for both the surgery and the pre- and post-operative assessments and follow-up. The choice between a longitudinal or transverse incision was based on patient preferences. Only patients with moderate to severe CTS who opted for surgical treatment were included in the study, while those with mild CTS were excluded to ensure minimal differences in baseline symptoms and grip strength during the preoperative period. Patient clinical findings, grip strength measurements using a JAMAR hydraulic hand dynamometer (Sammons Preston Rolyan, Bolingbrook, IL, USA), and Boston Carpal Tunnel Syndrome Questionnaire (BCTQ) scores were recorded at various time points: pre-operative (T0), one month (T1), three months (T2), and six months (T3) following the intervention. Our study included Lebanese individuals aged between 18 and 80 years old who were clinically diagnosed with moderate to severe CTS and confirmed as such through nerve conduction studies. These individuals were willing to undergo mini-open CTR using either a mini transverse or longitudinal incision. Excluded from the study were individuals with a history of acute wrist trauma or deformity, systemic illnesses that could impact the carpal tunnel (such as diabetes, thyroid dysfunction, autoimmune disorders), pregnant women, and those who had previously undergone wrist and forearm surgery.

All patients were operated on by the same senior orthopedic surgeon (R.M.) under locoregional anesthesia with the patient in the supine position. The tourniquet was inflated to 250 mmHg. Parenteral cefazolin was administered prophylactically preoperatively. The wrist was placed in mild extension using a lead hand.

In the longitudinal palmar limited incision technique, a two-centimeter skin incision was made at the intersection of the Kaplan cardinal line and the radial border of the fourth ray just distal to the wrist crease. ﻿Subcutaneous tissue was bluntly dissected until the flexor retinaculum was reached. Division of the latter was performed directly under visualization, aligning with the skin incision. Verification of median nerve release was conducted both proximally and distally. Wound irrigation was performed and the skin was closed using a non-absorbable monofilament suture.

**Figure 1:**
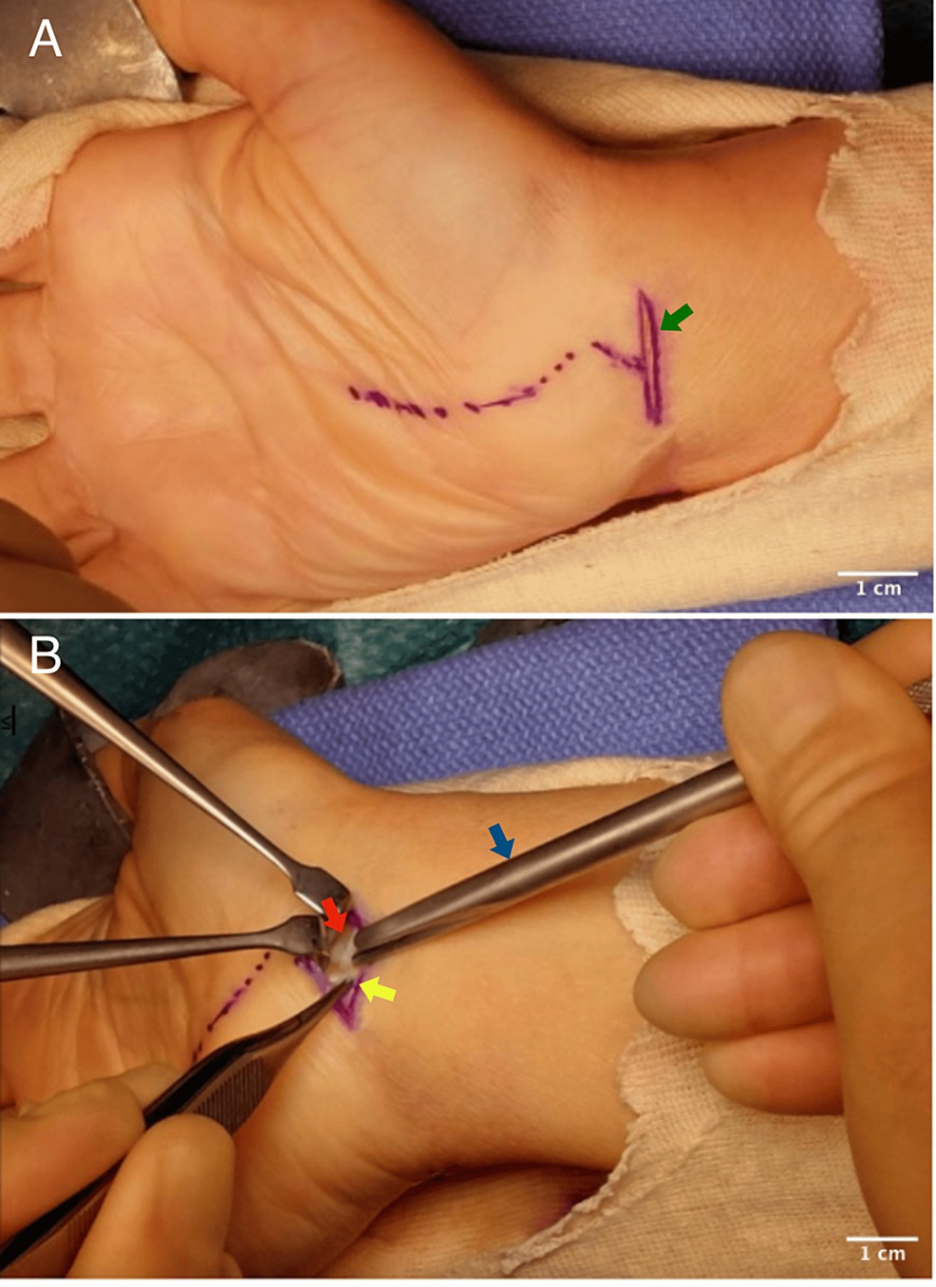
Image A: Green arrow showing the transverse incision at the wrist crease; Image B showing the cutting of the proximal edge of the flexor retinaculum (red arrow) using the Hagan carpel tunnel release sleeve (blue arrow) and the median nerve (yellow arrow) before its entry into the carpal tunnel.

In the mini-invasive transverse flexor crease incision technique, a short two centimeters transverse incision was made at the distal wrist (Figure 1A) crease midway between flexor carpi radialis (FCR) and flexor carpi ulnaris (FCU) tendons. The palmaris longus tendon was identified and retracted radially. The antebrachial fascia was incised and the proximal edge of the flexor retinaculum was exposed. A longitudinal incision was made on the proximal edge of the flexor retinaculum, revealing the median nerve. Using a Hagan CTR sleeve (Figure 1B), the transverse carpal ligament was released to its distal end. The cut is directed towards the third intermetacarpal space, gradually cutting the flexor retinaculum until the tissue resistance suddenly gives way indicating a complete division of the ligament. Wound irrigation was performed and the skin was closed using a non-absorbable monofilament suture.

The BCTQ Questionnaire was used to analyze the overall functional status and symptom severity pre- and post-operatively. Grip strength was measured using a Jamar Dynamometer and reported in kg.

Statistical Package for the Social Sciences (IBM SPSS Statistics for Windows, IBM Corp., Version 21.0, Armonk, NY) was used in the univariable and multivariable data analysis and management to compare the longitudinal CTR with the minimally invasive transverse incision technique. Descriptive statistics were reported as mean and standard deviation for continuous variables. Independent sample T-test and Mann-Whitney U test were used to determine if our findings were statistically valid. P-value <0.05 was considered as statistically significant. The Kolmogorov-Smirnov (KS) test was used to verify the normal data distribution.

## Results

**Figure 2:**
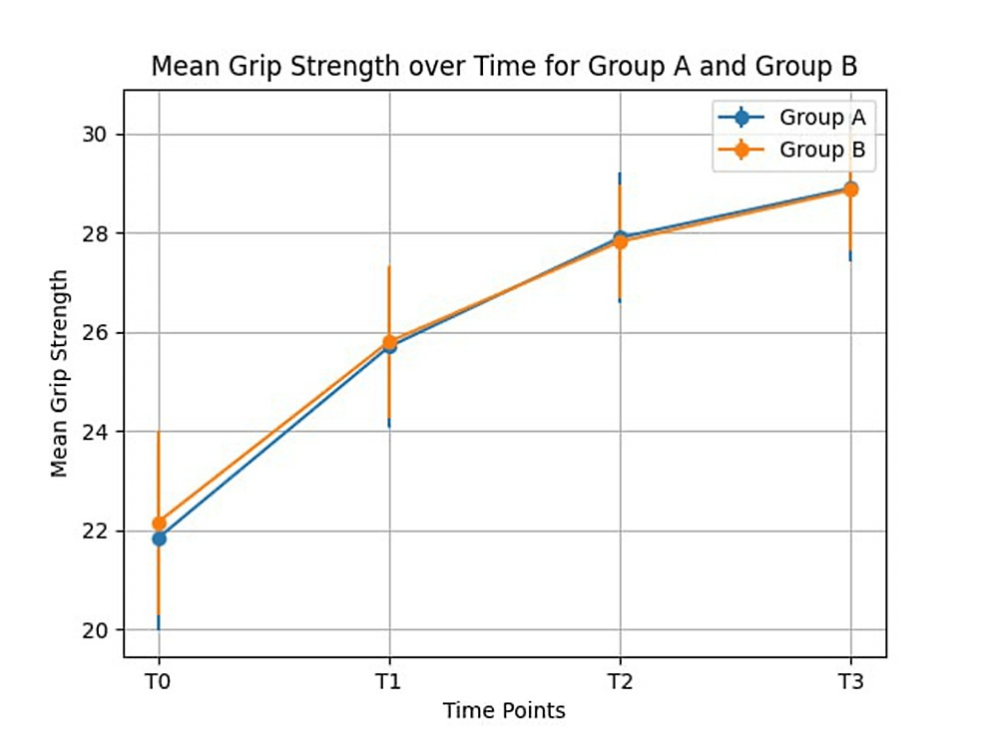
Graphic representation of the evolution of the grip strength (measured in kg) in the pre- and post-operative period in groups A and B

A total of 415 patients met the criteria of our research subject. Independent sample T-test and Mann-Whitney U Test were used to compare the mean of both groups. T-test was used for SSS (symptoms severity score) because the KS test showed normal data distribution in the mini-open transverse CTR (p=0.20>0.05) and limited palmar longitudinal CTR (p=0.17>0.05) for the other variables U test was used. Group A (longitudinal palmar mini-incision) included 221 individuals and Group B (transverse incision at the wrist crease) included 194 individuals. From Group A one patient lost to follow-up at one month post-op (post-operation), two patients at three months post-op, and three patients at six months post-op. From Group B two patients lost to follow-up at one month and three months post-op and three patients at six months post-op. All patients included in our study had positive Tinel’s and Phalen’s signs and the nerve conduction study showed moderate/severe CTS. The baseline demographic (shown in Table 1) was with no significant difference between the two groups. All patients showed improvement in grip strength at T1 (18% of group A vs 14% of group B), T2 (12% of group A vs 14% of group B), and T3 (3% of group A vs 4% of group B). Overall improvement in grip strength was similar with group A grip strength increasing by 33% and group B grip increasing by 32% with no statistically significant difference between the two. Results of grip strength are shown in Table 2 and represented by a graph in Figure 2. BCTQ score has improved from the pre- to postoperative period in both groups with no significant differences between the two techniques. FSS (functional severity score) improved with an average of 13 points and SSS improved with an average of 17 points. Detailed results are shown in Table 3.

**Table 1:** Patients’ basic demographic data

	Group A (n = 221)	Group B (n= 194)	P value
Age (Mean ± SD)	58.4 ± 5.1	58.8 ± 4.8	0.79
Gender			0.10
female	76.7% (n=168)	70.1% (n=136)	
male	22.8% (n=50)	29.9% (n=58)	

**Table 2:** Grip strength, with the grip strength value mean in (kg) and their associated standard deviation

	Group A (n = 221)	Group B (n= 194)	P value
GS at T0 (Mean ± SD)	21.84 ± 1.87	22.16 ± 1.86	0.09
GS at T1 (Mean ± SD)	25.70 ± 1.62	25.80 ± 1.54	0.46
GS at T2 (Mean ± SD)	27.90 ± 1.32	27.82 ± 1.16	0.62
GS at T3 (Mean ± SD)	28.90 ± 1.48	28.86 ± 1.21	0.53

**Table 3:** BCTQ score (functional severity score (FSS) and symptoms severity score (SSS)) reported as mean and standard deviation in the postoperative and at one month, three months, and six months postoperative. BCTQ: Boston Carpal Tunnel Syndrome Questionnaire

	Group A (n = 221)	Group B (n= 194)	P value
SSS T0 (Mean ± SD)	33.70 ± 6.39	32.82 ± 6.17	0.11
FSS T0 (Mean ± SD)	28.43 ± 4.31	28.92 ± 3.85	0.89
SSS T1 (Mean ± SD)	22.10 ± 3.39	19.96 ± 2.92	0.28
FSS T1 (Mean ± SD)	17.84 ± 2.89	19.15 ± 16.48	0.20
SSS T2 (Mean ± SD)	17.07 ± 4.14	16.48 ± 3.03	0.52
FSS T2 (Mean ± SD)	15.72 ± 2.89	16.85 ± 2.74	0.07
SSS T3 (Mean ± SD)	16.01 ± 2.50	15.26 ± 3.00	0.97
FSS T3 (Mean ± SD)	14.40 ± 2.91	15.11 ± 2.75	0.87

The only major complication was the development of pseudoaneurysm two months following CTR using the transverse incision at the wrist crease, and it was due to injury to the superficial palmar arch the patient was operated on for excision of the pseudoaneurysm; this rare complication was reported as a separate case report [8]. Other minor complications that were encountered using the transverse incision CTR were ecchymosis at the hand and forearm in three patients, and transient postoperative neuropraxia of the median nerve in one case that resolved on two weeks follow-up. Complications status post-CTR using limited palmar longitudinal incision was ecchymosis at the palmar region in two cases, erythema at the surgical wound in three cases that were treated prophylactically with antibiotics with complete resolution, two patients reported paresthesia at the incision site at a one-month follow-up.

## Discussion

CTS constitutes a significant portion of neuropathies affecting the upper limb. In this study, we aimed to investigate the clinical outcomes and patients’ satisfaction in patients who did open mini longitudinal incision versus transverse mini-invasive CTR. The major highlights of our studies are the large sample size (N = 480) in relation to other studies on the subject [9-11] and the fact that our study compares transverse mini-incision at the wrist crease to longitudinal mini-incision at the base of the palmar region while other studies compare transverse incision CTR to traditional open CTR [11]. Furthermore, our patients were followed and compared at regular intervals in time in the pre- and post-operative period and evaluated at each period in the post-operative period at one, three, and six months post operatively using a standardized scoring system for patients’ satisfaction and hand grip dynamometer to quantify the pre- and post-operative results in an objective and scientific manner while other studies on the subject did not apply the same systematic methodology [12]. While there are some studies that showed that mini-longitudinal incision is more effective on symptom and functional conditions than transverse incision [10], our studies showed no statistically significant difference between the two groups in terms of patients' satisfaction and functional outcome. The fact that only one major complication (a pseudoaneurysm caused by injury to the superficial palmar arch) occurred among the 221 patients who underwent CTR with a transverse incision at the wrist crease emphasizes the safety of this technique. This finding may encourage more surgeons to consider it as their primary approach for CTR. Traditional open CTR, while allowing for neurolysis and direct visualization of the median nerve, is not without its drawbacks. Kluge et al. have highlighted the main disadvantages, including pillar pain, tender scars, and delayed return to work [13]. Conversely, Serra et al. in a study of 112 individuals demonstrated that using a mini-invasive technique was associated with a smaller scar, less postoperative pain, and a quicker postoperative recovery [14]. Moreover, multiple studies have favored the transverse incision over the longitudinal incision due to its shorter healing time and smaller scar [15-17]. In comparison to endoscopic CTR using the same transverse incision, our mini-open technique offers advantages. Endoscopic methods carry a higher risk of iatrogenic injury to vital anatomical structures like injury to the ulnar artery, median nerve serious lacerations, and flexor tendon divisions [18] and are associated with increased costs due to the use of specialized equipment. Additionally, the endoscopic technique is known to have a steep learning curve, which has contributed to its limited use [19]. The mini-open technique's cost-effectiveness and accessibility to a broader population make it a compelling choice. Our study's strengths lie in its prospective follow-up, standardized scoring system (BCTQ scoring), and objective grip strength measurements using the same dynamometer. Furthermore, surgeries were consistently performed by a skilled senior orthopedic surgeon (R.M.) at the same institution (CMC) and our study has only included adult Lebanese patients, minimizing confounding factors related to ethnicity, surgical technique, or materials used. The larger sample size enhances the power of our analysis. Notably, data collection via the BCTQ questionnaire was conducted by senior orthopedic residents at CMC to prevent potential misinterpretation of the questions. However, some limitations should be acknowledged, including a few patients lost to follow-up, resulting in an incomplete dataset. The post-operative follow-up period was limited to six months, and data on patients' smoking status were unavailable.

## Conclusions

In conclusion, our study provides valuable insights into the clinical outcomes and patient satisfaction associated with transverse mini-invasive CTR compared to the longitudinal CTR mini-incision. The distinctive aspects of our research lie in several aspects. Firstly, we conducted a comprehensive comparison of two mini-open CTR techniques with a large sample size (N = 480), providing robust statistical power. Secondly, our study uniquely focused on comparing the transverse incision technique at the wrist crease to the longitudinal incision technique at the base of the palmar region, which is a less common comparison in the literature. Additionally, our systematic methodology, including regular pre- and post-operative assessments using standardized scoring systems and objective grip strength measurements, adds to the scientific rigor of our findings. The safety and efficacy of the transverse incision technique observed in our study support its consideration as a primary surgical approach for CTR. Compared to traditional open CTR and endoscopic techniques, the mini-open transverse incision offers advantages such as shorter healing time, smaller scars, and lower risk of iatrogenic injuries. Moreover, its cost-effectiveness and accessibility make it an attractive option for surgeons and patients alike. Overall, our findings contribute to the growing body of evidence supporting the use of the mini-open transverse incision technique for CTR, offering surgeons a safe and effective alternative to traditional open and endoscopic approaches.
